# O acesso de crianças com síndrome congênita pelo Zika vírus às políticas públicas

**DOI:** 10.1590/0102-311XPT014624

**Published:** 2025-02-07

**Authors:** Ana Cristina Pannain L. Kaufman, Andre Reynaldo Santos Périssé, Cristina Barroso Hofer

**Affiliations:** 1 Instituto de Puericultura e Pediatria Martagão Gesteira, Universidade Federal do Rio de Janeiro, Rio de Janeiro, Brasil.; 2 Ministério da Previdência Social, Rio de Janeiro, Brasil.; 3 Escola Nacional de Saúde Pública Sergio Arouca, Fundação Oswaldo Cruz, Rio de Janeiro, Brasil.; 4 Departamento de Doenças Infecciosas e Parasitárias, Universidade Federal do Rio de Janeiro, Rio de Janeiro, Brasil.

**Keywords:** Microcefalia, Vírus da Zika, Políticas Públicas, Microcephaly, Zika Virus, Public Policies, Microcefalia, Virus Zika, Políticas Públicas

## Abstract

A síndrome congênita da Zika (SCZ) é responsável por várias malformações, inclusive microcefalia. Os objetivos deste estudo foram: descrever o acesso das crianças às políticas públicas sociais de pensão vitalícia (E60), criada frente à emergência de saúde pública do Zika vírus (Espin-ZIKV), e ao Benefício de Prestação Continuada/Espécie 87 (BPC/E87), e comparar esse acesso antes e depois da Espin-ZIKV. Estudo transversal, com extração e descrição de dados do Sistema de Informações sobre Nascidos Vivos (SINASC), Registro de Eventos em Saúde Pública-Microcefalia (Resp-Microcefalia) e Sistema Único de Informação de Benefícios (SUIBE), entre 2013 e 2021. Descrevemos as E60 que ainda estavam ativas quando da extração de dados do SUIBE em maio de 2023. Comparamos a concessão do BPC/E87 por microcefalia em 2013 e 2021 (antes e depois da Espin-ZIKV). Dos 20.000.859 nascidos vivos entre 2015-2021, foram notificados 20.464 casos suspeitos de SCZ no Resp-Microcefalia; 20% dos pacientes acometidos receberam algum benefício social: 705 a E60 e 3.822 o BPC/E87. A média nacional de concessão de BPC/E87 em 2013 por microcefalia foi de 8 para cada 100 mil nascidos vivos (antes da Espin-ZIKV) e 5 BPC/E87 para cada 100 mil nascidos vivos em 2021 (após Espin-ZIKV) (p < 0,01). Observamos 689 crianças com E60 ativas (entre 3 e 8 anos de idade) em maio de 2023. O estudo evidenciou baixa proteção social das crianças com suspeita de SCZ, mas, dentre as que se beneficiaram da E60, 98% estavam com benefício ativo em 2023, demonstrando sobrevida importante das crianças beneficiadas. Foi observada redução na média de concessões de BPC/E87 para microcefalia sem causa especificada após Espin-ZIKV.

## Abstract (en)

Congenital Zika syndrome (CZS) is responsible for several malformations, including microcephaly. This study aimed to describe children’s access to social public policies: lifetime benefit (E60) created in the face of the Zika virus public health emergency (Espin-ZIKV) or Continuous Cash Benefit/Type 87 (BPC/E87). Thus, this study compares the children’s access before and after Espin-ZIKV. This was a cross-sectional study, with data extraction and description from the Brazilian Information System on Live Births (SINASC), the Registry of Public Health Events-Microcephaly (Resp-Microcephaly), and the Unified System for Benefits Information (SUIBE), from 2013 to 2021. We describe the E60 that were still active at the time of SUIBE data extraction in May, 2023. We compared the granting of BPC/E87 for microcephaly in 2013 and 2021 (before and after Espin-ZIKV). Of the 20,000,859 live births from 2015-2021, 20,464 suspected cases of CZS were reported in Resp-Microcephaly; 20% of affected patients received some social benefit: 705 received E60 and 3,822 received BPC/E87. The national average of BPC/E87 granted in 2013 for microcephaly was eight for every 100,000 live births (before Espin-ZIKV), while in 2021 it was five BPC/E87 for every 100,000 live births (after Espin-ZIKV) (p < 0.01). We observed 689 children with active E60 (from 3 to 8 years of age), in May, 2023. The study showed low social protection for children with suspected CZS. However, of those who benefited from the E60, 98% had active benefits in 2023, demonstrating the important survival of the benefited children. A reduction in the mean number of BPC/E87 grants for microcephaly without specified cause was observed after Espin-ZIKV.

## Abstract (es)

El síndrome congénito del Zika (SCZ) es responsable de varias malformaciones, incluida la microcefalia. Los objetivos de este estudio fueron: describir el acceso de los niños a las políticas públicas sociales -pensión vitalicia (E60) creada en respuesta a la emergencia de salud pública provocada por el virus del Zika (Espin-ZIKV) o Beneficio de Prestación Continuada/Especie 87 (BPC/E87)- y comparar este acceso antes y después de Espin-ZIKV. Estudio transversal, con extracción y descripción de datos del Sistema de Informaciones sobre Nacidos Vivos (SINASC), Registro de Eventos en Salud Pública-Microcefalia (Resp-Microcefalia) y Sistema Único de Información de Beneficios (SUIBE), entre el 2013 y el 2021. Describimos las E60 que todavía estaban activos cuando se extrajeron los datos de SUIBE en mayo del 2023. Comparamos la concesión del BPC/E87 por microcefalia en el 2013 y el 2021 (antes y después de Espin-ZIKV). De los 20.000.859 nacidos vivos entre 2015-2021, se notificaron 20.464 casos sospechosos de SCZ en Resp-Microcefalia; el 20% de los pacientes afectados recibió algún beneficio social: 705 la E60 y 3.822 recibieron BPC/E87. El promedio nacional de otorgamiento de BPC/E87 en el 2013 para microcefalia fue de ocho por cada 100.000 nacidos vivos (antes de Espin-ZIKV) y de 5 BPC/E87 por cada 100.000 nacidos vivos en el 2021 (después de Espin-ZIKV) (p < 0,01). Observamos 689 niños con E60 activas (entre 3 y 8 años) en mayo del 2023. El estudio puso de manifiesto una baja protección social para los niños con sospecha de SCZ, pero, de los que se beneficiaron de la E60, el 98% tenía beneficios activos en el 2023, lo que demuestra una importante sobrevida de los niños beneficiados. Se observó una reducción en el promedio de subvenciones de BPC/E87 para microcefalia sin causa especificada, tras Espin-ZIKV.

## Introdução

A microcefalia, associada ao comprometimento do desenvolvimento cerebral e neuropsicomotor, pode ser decorrente de fatores genéticos e fatores ambientais, como exposição intrauterina ao álcool, drogas, radiação e infecções durante a gestação ^1^ ^,^ ^2^. A infecção pelo Zika vírus (ZIKV) durante o período intraútero pode levar a diversas malformações para o concepto, como alterações no sistema nervoso central, crescimento intrauterino retardado (CIUR), baixo peso ao nascer, excesso de pele nucal, anormalidades visuais e auditivas, irritabilidade, crises convulsivas, disfagia, hipertonia ou hipotonia, discinesias, hemiplegia, hemiparesia, espasticidade e, com menos frequência, artrogripose (contratura articular) e pés tortos. O conjunto dessas malformações foi denominado síndrome congênita da Zika (SCZ) ^3^ ^,^ ^4^.

O aumento expressivo dos casos de microcefalia/SCZ associada à infecção de gestantes pelo ZIKV fez com que o Ministério da Saúde declarasse estado de emergência em saúde pública de importância nacional (Espin) em novembro de 2015, com a inclusão da SCZ às causas comuns de microcefalia ^1^ ^,^ ^5^.

As deficiências nas redes básicas de saúde e saneamento, em especial nas comunidades carentes e centros urbanos, aumentam o risco da infecção congênita pelo ZIKV na população econômica mais vulnerável ^6^ ^,^ ^7^ ^,^ ^8^. A desestruturação do núcleo familiar, em uma população já em alta vulnerabilidade, é frequentemente observada no cuidado de famílias de crianças com SCZ. A sobrecarga laboral do responsável, na grande maioria das vezes a mãe, vivenciada pelo cuidado de uma criança com importante comprometimento global, junto com dificuldades econômicas, alteração da dinâmica familiar e desamparo social, contribui para essa realidade ^9^ ^,^ ^10^. Como consequência, é frequentemente observado o adoecimento psíquico dessas mães ^10^ ^,^ ^11^ ^,^ ^12^ ^,^ ^13^ ^,^ ^14^.

Os custos decorrentes dos cuidados com as crianças com microcefalia como sequela da disseminação do ZIKV se somam aos cuidados sociais, gerando ônus imediato sobre os sistemas de saúde e bem-estar social e impactos econômicos a médio e longo prazo ^15^ ^,^ ^16^.

Ações emergenciais para fortalecer a atenção à saúde e proteção social de crianças com microcefalia foram instituídas pela *Medida Provisória nº 712/2016* (MP 712/2016) ^17^, pela *Portaria Interministerial nº 405/2016* ^18^ e pela *Lei nº 13.301/2016* ^19^, que determinaram a concessão do Benefício de Prestação Continuada/Espécie 87 (BPC/E87), já existente, no valor de um salário mínimo vigente, a crianças acometidas pela SCZ cujas famílias apresentavam critério de vulnerabilidade econômica (renda *per capita* inferior a ¼ do salário mínimo vigente), com a manutenção do benefício até os três anos de vida das crianças ^19^ ^,^ ^20^.

O governo do Brasil, após deliberações sobre a eficiência das medidas iniciais, por meio da MP 894/2019 ^21^ ^,^ ^22^, criou uma pensão indenizatória mensal, vitalícia e intransferível a cargo da União, no valor de um salário mínimo vigente, destinada às crianças com microcefalia decorrente do ZIKV já beneficiárias do BPC/E87 nascidas no período entre 1º de janeiro de 2015 e 31 de dezembro de 2018, posteriormente ampliado para até 31 de dezembro de 2019, e designada como Espécie 60 (E60) pela *Portaria nº 66/2020* do Instituto Nacional do Seguro Social (INSS) ^23^. A *Emenda Modificativa nº 113/2019* ^21^, de forma diversa do BPC/E87, estabeleceu abono anual e pensão por morte com duração de até 12 meses para o responsável civil pela criança que comprovasse dedicação exclusiva a ela e recebesse a Pensão Vitalícia por SCZ (E60), além da prorrogação da licença-maternidade e salário-maternidade por 180 dias para as mães de filhos com SCZ.

A MP 894/2019 foi criticada por limitar a data de nascimento das crianças com direito ao recebimento da pensão e por incluir as crianças que já recebiam o BPC/E87 por sequelas neurológicas da Zika congênita com nascimento no mesmo período. A *Portaria nº 66/2020* estabeleceu em 30 de janeiro de 2020 as regras e os procedimentos para requerimento e concessão da E60, com a operacionalização pelo INSS, a troca da nomenclatura de microcefalia do ZIKV para SCZ e a ampliação do prazo de nascimento para as crianças com SCZ nascidas no período de 1º de janeiro de 2015 a 31 de dezembro de 2019 ^23^.

A *Lei nº 13.985/2020* ^24^ instituiu pensão especial destinada a crianças com SCZ nascidas entre 1º de janeiro de 2015 e 31 de dezembro de 2019 e determinou os critérios para concessão da E60. Associado ao critério de renda *per capita* inferior a ¼ do salário mínimo vigente ^19^, haveria necessidade de avaliação por perito médico federal para constatação da relação entre as alterações apresentadas pela criança e a infecção pelo ZIKV, conforme critérios do Ministério da Saúde publicados no documento *Orientações Integradas de Vigilância e Atenção à Saúde no Âmbito da Emergência de Saúde Pública de Importância Nacional* ^25^.

O objetivo do presente estudo foi descrever, entre as crianças nascidas no Brasil no período de 1º de janeiro de 2013 a 31 de dezembro de 2021, a dinâmica de acesso a benefícios sociais, seja a E60 entre as crianças com o diagnóstico de SCZ nascidas entre 2015 e 2019 ou o BPC/E87. Entre as crianças que receberam a E60, descreveremos também a atividade do benefício, até maio de 2023. Um segundo objetivo do estudo é comparar o acesso ao BPC/E87 das crianças com microcefalia sem causa especificada nascidas antes (2013) e das nascidas após a Espin-ZIKV (2021).

## Material e métodos

**Figura 1:**
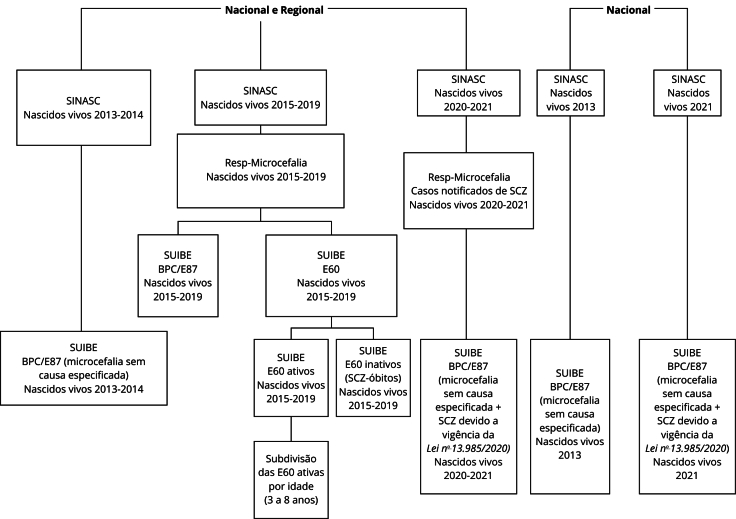
Fluxograma da metodologia empregada no estudo.

Estudo transversal, por meio da coleta de dados das bases de sistemas do Ministério da Saúde e do INSS referentes ao período de 2013 e 2021. O período de estudo entre 2013 e 2021 foi escolhido a fim de descrever as concessões de E60 (para crianças com SCZ nascidas durante a vigência da *Lei nº 13.985/2020*) e de BPC/E87 por microcefalia em geral, antes, durante e após a Espin-ZIKV (Figura 1).

BPC/E87: Benefício de Prestação Continuada também descrito como Espécie 87; E60: pensão vitalícia destinada a crianças com SCZ; Resp-Microcefalia: Registro de Eventos em Saúde Pública-Microcefalia; SCZ: síndrome congênita pelo Zika vírus; SINASC: Sistema de Informações sobre Nascidos Vivos; SUIBE: Sistema Único de Informação de Benefícios.

A população de estudo incluiu os nascidos vivos registrados no Sistema de Informações sobre Nascidos Vivos (SINASC) ^26^, os casos notificados como suspeitos de SCZ constantes no Registro de Eventos em Saúde Pública-Microcefalia (Resp-Microcefalia) ^27^, plataforma de notificação para vigilância epidemiológica de microcefalias/SCZ associadas ao ZIKV do Sistema Único de Saúde (SUS) e Ministério da Saúde, e as crianças com microcefalia que receberam alguma espécie de benefício conforme registro no Sistema Único de Informação de Benefícios (SUIBE), sistema de acesso restrito do INSS.

O SINASC é um dos sistemas de informação em saúde criado pelo Ministério da Saúde e gerido pela Secretaria de Vigilância em Saúde e Ambiente (SVSA) em que são coletados dados sobre nascidos vivos, por meio da Declaração de Nascido Vivo (DNV).

O Resp, formulário desenvolvido pelo Departamento de Informática do SUS (DATASUS), tem por finalidade registrar as emergências em saúde pública e possibilitar a análise rápida da situação epidemiológica e a adoção de medidas emergenciais. O aumento do número de casos de microcefalia e de alterações do sistema nervoso central que ocorreu durante a epidemia de ZIKV ensejou a criação do Resp-Microcefalia em 15 de novembro de 2015, a fim de identificar os fatores associados ao aumento do número de nascimentos de crianças com microcefalia e outras alterações neurológicas descritas em 2015 ^28^. Os casos registrados no sistema Resp-Microcefalia foram divididos entre casos suspeitos e casos confirmados. O nosso estudo analisou os casos registrados como suspeitos de SCZ, pois estes foram os casos avaliados pelo INSS para concessão da E60.

O SUIBE do INSS contém informações de todos os benefícios solicitados, deferidos ou não. Os benefícios são divididos em vários tipos, designados como “espécies” de benefícios, e recebem uma numeração individual para cada requerente. O SUIBE surgiu da necessidade de criar um banco único de dados contendo todas as funções, como solicitação, concessão, pagamento, manutenção e atualização de benefícios, o que propiciou ações gerenciais e a minimização de erros anteriores quando da presença de vários sistemas que apresentavam dificuldade de intercomunicação ^29^.

As crianças portadoras de microcefalia que receberam algum benefício foram subdivididas em dois grupos: as que receberam o BPC/E87 por apresentarem microcefalia sem causa especificada e aquelas que receberam a E60. As concessões de BPC/E87 e de E60 devido a SCZ foram descritas e estudadas a partir da data de nascimento das crianças para evitar que, devido ao lapso de tempo entre o nascimento das crianças e a concessão dos auxílios, ocorresse um viés de interpretação temporal dos resultados apresentados.

O estudo descreveu os nascidos vivos registrados no SINASC entre 2013 e 2021 (inclusive), os casos notificados de SCZ no Resp-Microcefalia até o final do período de estudo, as crianças com microcefalia/SCZ que conseguiram ser beneficiadas pela E60 e aquelas que receberam o BPC/E87 por microcefalia sem causa especificada, ambas registradas no SUIBE. Os dados foram comparados nos âmbitos nacional e regional, com descrição das concessões do BPC/E87 e de E60 distribuídos em três períodos, entre 2013 e 2014 (antes da Espin-ZIKV), entre 2015 e 2017 (durante Espin-ZIKV) e entre 2018 e 2021 (após Espin-ZIKV). A idade das crianças com E60 ativa, no momento da extração desses dados (4 de maio de 2023), também foi estudada no Brasil e suas macrorregiões (Figura 1).

A ausência ou presença de dados no DATASUS sobre causas não infecciosas ou algumas causas infecciosas de microcefalia, assim como a presença, por meio da 10ª revisão da Classificação Internacional de Doenças (CID-10), sob a designação de CID Q02 (microcefalia), no setor de anomalia ou defeito congênito em nascidos vivos no SINASC ^30^, determinou a análise dos casos de microcefalia por meio dos dados disponíveis no Resp-Microcefalia (criado em novembro de 2015).

Para avaliar o acesso aos benefícios, frente à Espin-ZIKV, utilizamos as variáveis: nascidos vivos no período de estudo; casos notificados como suspeitos de infecção congênita pelo ZIKV (registrados no Resp-Microcefalia durante o período de estudo); os beneficiários do BPC/E87 por microcefalia em geral (nascidos entre 2013 e 2021) ou que receberam a E60, referente às crianças nascidas entre 2015 e 2021, conforme estabelecido na *Lei nº 13.985/2020* (Figura 1).

Para comparar o acesso à E87 por microcefalia antes e após a Espin-ZIKV, foi calculado o número de crianças nascidas por ano que receberam BPC/E87 por 100 mil nascidos vivos, utilizando o número de BPC/E87 concedidos por microcefalia (de acordo com o SUIBE) no ano estudado como numerador e o número de nascidos vivos no mesmo ano (de acordo com o SINASC) como denominador, transformando em 100 mil nascidos vivos.

As variáveis foram estudadas e, como apresentavam distribuição normal, utilizamos o teste t de Student, bicaudal para a comparação das médias. A significância estatística foi considerada quando p < 0,05.

O estudo foi iniciado após aprovação pelo Comitê de Ética em Pesquisa do Instituto de Puericultura e Pediatria Martagão Gesteira, Universidade Federal do Rio de Janeiro (CAAE nº 41089920.5.0000.5264 e parecer nº 4.492.130, aprovado em 7 de janeiro de 2021), dispensado o Termo de Consentimento Livre e Esclarecido pelo uso de dados públicos constantes no SINASC e Resp-Microcefalia e com autorização expressa pela Secretaria de Perícia Médica Federal para utilização dos dados do SUIBE. Todos os dados utilizados foram anônimos, e os autores não tiveram acesso a nenhum dado que pudesse identificar qualquer participante do estudo.

## Resultados

O estudo demonstrou que, antes de Espin-ZIKV (entre 2013 e 2014), a natalidade foi de 5.883.286 (2.941.643 nascidos vivos por ano), com a concessão de 473 BPC/E87 para nascidos vivos no período (236,5 BPC/E87 para nascidos vivos por ano). O período de Espin-ZIKV, entre 2015 e 2017, registrou 8.799.535 nascidos vivos (2.933.178 por ano), 15.380 casos notificados de SCZ, 2.452 (817,4 por ano) concessões de BPC/E87 e 679 (226,4 por ano) concessões de E60 em nascidos vivos durante o período no país. Após a Espin-ZIKV, foram registrados 11.201.324 nascidos vivos (2.800.331 por ano), 5.083 casos de SCZ e 897 BPC/E87 (224,2 BPC/E87 por ano). Foram aprovadas 26 concessões de E60 em nascidos vivos entre 2018 e 2019, quando finalizou o prazo de vigência da *Portaria nº 66/2020* (que estabeleceu o direito a concessão de E60 para nascidos vivos com SCZ entre 2015 e 2019) (Tabela 1).

**Tabela 1:** Natalidade, casos notificados de síndrome congênita de Zika (SCZ), Benefício de Prestação Continuada/Espécie 87 (BPC/E87) e pensão vitalícia decorrente da SCZ (E60) - antes, durante e após epidemia de ZIKV.

	2013-2014	2015-2017	2018-2021	Período do estudo (2013-2021)
Nacional				
Nascidos vivos	5.883.286	8.799.535	11.201.324	25.884.145
Casos notificados de SCZ	-	15.380	5.084	20.464
BPC/E87	473	2.452	897	3.822
E60	-	679	26	705
Região Nordeste				
Nascidos vivos	2.332.006	2.459.804	3.179.267	7.971.077
Casos notificados de SCZ	-	9.302	1.856	11.158
BPC/E87	161	1.274	365	1.800
E60	-	500	16	516
Região Sudeste				
Nascidos vivos	1.655.495	3.475.563	4.312.462	9.443.520
Casos notificados de SCZ	-	3.684	2.015	5.699
BPC/E87	189	625	251	1.065
E60	-	100	1	101
Região Norte				
Nascidos vivos	633.052	941.132	1.243.056	2.817.240
Casos notificados de SCZ	-	1.048	329	1.377
BPC/E87	44	264	88	396
E60	-	23	3	26
Região Sul				
Nascidos vivos	782.860	1.195.923	1.519.697	3.498.480
Casos notificados de SCZ	-	419	384	803
BPC/E87	51	118	45	214
E60	-	8	2	10
Região Centro-oeste				
Nascidos vivos	479.609	726.581	946.842	2.153.032
Casos notificados de SCZ	-	1.124	405	1.529
BPC/E87	32	165	72	269
E60	-	39	4	43

Nota: não constou no Sistema Único de Informação de Benefícios (SUIBE) a especificação da região do país onde 10 casos de BPC/E87 e 8 de E60 foram concedidas.

**Figura 2:**
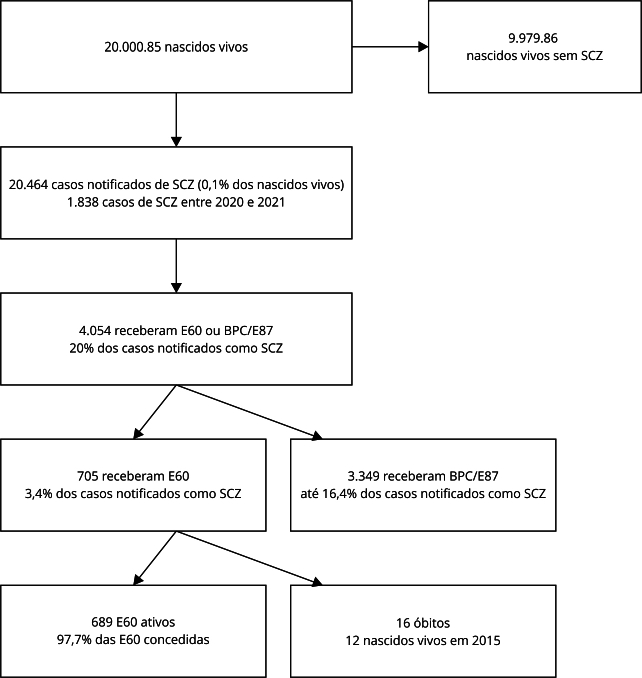
Concessão de pensão vitalícia decorrente da síndrome congênita de Zika (SCZ) (E60) e Benefício de Prestação Continuada/Espécie 87 (BPC/E87) entre 2015 e 2017 (durante a emergência em saúde pública de importância nacional pela epidemia de Zika vírus (Espin-ZIKV) e entre 2018 e 2021 (após Espin-ZIKV).

Observamos que, entre os nascidos vivos no Brasil, de 2015 a 2021 (n = 20.000.859), foram notificadas 20.464 (0,1%) suspeitas de SCZ, sendo que 4.054 (20%) da população notificada teve acesso a algum benefício (Figura 2), 3.131 durante a Espin-ZIKV e 923 após a Espin-ZIKV (Tabela 1).

As crianças com SCZ nascidas em 2020 e 2021 (n = 1.838), que correspondem a 9% dos casos de SCZ do estudo, não tiveram direito ao recebimento de E60 (Figura 2).

O Nordeste do Brasil foi a região que concentrou mais casos de SCZ (n = 9.302), assim como a concessão de BPC/E87 (n = 1.274) e E60 (n = 500) para nascidos vivos entre 2015 e 2017. A Região Sudeste, entre 2018-2021, passou a apresentar número de casos de SCZ (n = 2.105) semelhante ao observado no Nordeste do país (n = 1.856), mantendo, contudo, menor concessão de BPC/E87 (n = 251) e E60 (n = 1) no mesmo período (Tabela 1). A abrupta redução nacional entre 2018-2021 de crianças nascidas que receberam E60 (n = 26 em relação às 679 concessões no período anterior), também ocorreu em âmbito regional (Tabela 1).

No Brasil, em média, 8 em cada 100 mil nascidos vivos receberam BPC/E87 (intervalo de 95% de confiança - IC95%: 7-9) em 2013, média que foi reduzida para 5 em 100 mil nascidos vivos em 2021 (IC95%: 4-6), uma redução estatisticamente significativa (p < 0,01). Quando analisamos as macrorregiões brasileiras, vemos que apenas as regiões Nordeste (10 por 100 mil nascidos vivos em 2013 e 6 por 100 mil nascidos vivos em 2021; p < 0,01) e Sudeste (7 por 100 mil nascidos vivos em 2013 e 5 por 100 mil nascidos vivos em 2021; p < 0,01) apresentaram redução de concessão do BPC/E87 estatisticamente significativa (Tabela 2).

**Tabela 2:** Concessões de Benefício de Prestação Continuada/Espécie 87 (BPC/E87) por microcefalia sem causa especificada a nível nacional e regional antes e após a epidemia de síndrome congênita de Zika (SCZ).

	Crianças nascidas em 2013 que receberam BPC/E87 por 100.000 nascidos vivos [n (IC95%)]	Crianças nascidas em 2021 que receberam BPC/E87 por 100.000 nascidos vivos [n (IC95%)]	Valor de p
Brasil	8 (7-9)	5 (4-6)	< 0,01
Região Nordeste	10 (8-12)	6 (5-8)	0,01
Região Sudeste	7 (6-9)	5 (3-6)	0,01
Região Centro-oeste	5 (2-8)	3 (0-5)	0,13
Região Norte	6 (3-9)	4 (2-7)	0,41
Região Sul	7 (5-10)	4 (2-6)	0,08

IC95%: intervalo de 95% de confiança.

Fonte: elaboração própria, a partir de dados extraídos do Sistema de Informações sobre Nascidos Vivos (SINASC) ^26^ e do Sistema Único de Informação de Benefícios (SUIBE).

O estudo mostrou a concessão de 705 E60 e, no momento da extração de dados do SUIBE, havia 689 E60 ativas em crianças entre 3 e 8 anos, com um total de 16 óbitos, 12 em crianças nascidas em 2015 (Figura 2). A maior parte das E60 ativas (n = 503; 73%) foi concedida em crianças nascidas no Nordeste do país, sendo 454 (90,2%) com 7 anos. Os demais grupos de crianças com E60 ativa corresponderam a 186 casos, 111 (59,7%) destas em crianças com 6 anos, sendo 67 (60,4%) no Sudeste do Brasil (Tabela 3).

**Tabela 3:** Pensões vitalícias decorrentes da síndrome congênita de Zika (SCZ) (E60) anual, nacional e regional, ativas em nascidos vivos no período de 2015 e 2019.

Região	Idade em 2023 (anos)						Total de E60 ativas
	3	4	5	6	7	8	
Nordeste	3	9	9	23	454	5	503
Sudeste	0	0	4	67	24	3	98
Norte	1	1	2	10	7	1	22
Sul	0	1	2	3	3	1	10
Centro-oeste	1	2	5	26	7	0	41
Sem registro	0	1	0	5	9	0	15
Nacional	5	14	22	134	504	10	689

Fonte: elaboração própria, a partir de dados do Sistema Único de Informação de Benefícios (SUIBE) em 4 de maio de 2023.

Nota: 705 E60 foram concedidas devido à SCZ, com 689 ativas e 16 óbitos até 4 de maio de 2023.

## Discussão

Neste estudo, compilamos dados de natalidade, notificações de pacientes com suspeita de SCZ, concessões de BPC/E87 de 2013 a 2021 e de E60 entre 2015 e 2019, por meio das bases de dados: SINASC, Resp-Microcefalia e SUIBE. De forma geral, nosso estudo demonstrou que, apesar do registro de um número expressivo de crianças com SCZ (n = 20.464 casos entre 2015-2021) no país, apenas cerca de 20% das crianças acometidas foram beneficiadas por alguma medida de seguridade social. O relato da presença de alta vulnerabilidade social em grande parte das famílias de crianças acometidas pelo ZIKV ^18^ sugere que esses benefícios não atingiram toda a população com direito aos mesmos. Ao mesmo tempo, o estudo considerou todos os pacientes nascidos entre 2015 e 2021 com quaisquer benefícios (BPC/E87 e E60) e por qualquer causa de microcefalia, o que pode ter causado superestimativa do número de benefícios por ZIKV, e a população beneficiada talvez seja ainda menor que os cerca de 20% estimados.

A diminuição observada na comparação da taxa de concessão de BPC/E87 dos anos de 2013 e 2021 (antes e depois da Espin-ZIKV) em todas as macrorregiões brasileiras, de forma estatisticamente significativa nas regiões Nordeste e Sudeste, sem que tenha ocorrido nenhum desenvolvimento científico ou social entre esses anos, aponta para a ausência de qualquer curva de aprendizado em relação aos direitos a benefícios das crianças com microcefalia após a Espin-ZIKV.

O registro do número de casos de SCZ no Brasil após 2019, ainda que tenha ocorrido uma grande redução das notificações, demonstrou a persistência dessa condição no país e a necessidade de atuar nos fatores de risco e manter os cuidados de saúde e proteção social à população mais vulnerável. A ausência de concessões de E60 após 2019 devido ao período de vigência da *Lei nº 13.985/2020*, e o registro em 2020 e 2021 de 1.838 casos de SCZ (9% do total de casos registrados durante o estudo), em especial no Sudeste e Nordeste do Brasil, demonstrou que estas crianças, por não terem direito à E60, receberam o BPC/E87 com a falta de proteção adequada da legislação específica quanto a pensão destinada a crianças com SCZ.

Estudo realizado por Pereira et al. ^31^, sobre o perfil da demanda de BPC/E87 concedido para crianças de até 48 meses diagnosticadas com microcefalia no Brasil entre o período de 2009 e 2016, observou uma média estável na concessão em torno de 200 benefícios por ano com uma redução destas em 2015 (atribuída a greve de peritos do INSS) e aumento de oito vezes na concessão em 2016. A análise realizada pelo ano da concessão do benefício pode levar a um viés na interpretação dos resultados, uma vez que analisa crianças com até 48 meses sem especificar o ano de nascimento. A data da concessão não reflete necessariamente coortes de nascimento, e, quando estudamos tais coortes, observamos o aumento na concessão de BPC/E87 em crianças nascidas entre 2015 e 2017, período que corresponde a Espin-ZIKV, em especial nas regiões Nordeste e Sudeste do país. A redução, por nós observada, na concessão de BPC/E87 para crianças nascidas entre 2018 e 2021, abaixo do período antes da Espin-ZIKV, e a ausência de ações ou estudos que demonstrem a redução de outras causas de microcefalia reforçam a ideia de falta de proteção adequada para esse grupo de pessoas, mesmo após a Espin-ZIKV vivenciada em nosso país.

Os trâmites necessários para concessão do BPC por microcefalia em geral e da pensão vitalícia devido a infecção congênita pelo ZIKV acarretam um lapso de tempo considerável entre o nascimento dessas crianças e o recebimento desses auxílios sociais ^32^. A taxa de letalidade para possíveis casos de SZC é de 10,4% e, entre 0 e 2 anos, 92,8% dos óbitos ocorrem em menores de um ano (52,6% no período neonatal e 40,2% no período pós-natal) ^33^. A partir dessas informações, podemos concluir que muitas dessas crianças não conseguiram receber auxílios, o que justifica a concessão de 705 pensões vitalícias pela SCZ no período estudado diante da Espin-ZIKV vivenciada no Brasil, em especial nos anos de 2015 e 2016. A presença de 689 casos de concessão de E60 ativas em 2023, comparado ao total de concessões entre 2015 e 2021 (n = 705), a maioria das crianças com 7 ou 8 anos em 2023 (nascidas em 2015 ou 2016, no auge da Espin-ZIKV), mostrou uma sobrevida alta das crianças beneficiadas frente aos comprometimentos por elas apresentados.

Estudo realizado por Sá et al. ^34^, sobre a dinâmica familiar das crianças com SCZ no Município de Petrolina (Pernambuco), demonstrou a necessidade de mudanças na rotina familiar para se adaptar à presença de uma criança que necessita de cuidados bem maiores que as demais. Houve aumento da demanda de cuidadores das crianças (em geral, a mãe) com desestruturação familiar, aliado à presença de cuidadores jovens, em idade produtiva, com baixa escolaridade e ausência de sistema de apoio. Os custos com cuidados das crianças com SCZ são elevados e podem comprometer a renda familiar pela necessidade de tratamento multidisciplinar e de permanência do representante legal nos cuidados diários de seus filhos e fora do mercado de trabalho formal, com redução da força de trabalho e agravamento da vulnerabilidade econômica destas famílias ^34^ ^,^ ^35^.

A ausência de dados no Resp-Microcefalia sobre as etiologias de microcefalia antes de 2015, infecciosas ou não, pode ter acarretado um viés de informação ao analisarmos os benefícios concedidos por microcefalia sem causa especificada (BPC/E87) e a pensão vitalícia pela SCZ (E60). A entrada do ZIKV no país, de acordo com estudo realizado por Campos et al. ^36^, ocorreu em 2014, e os casos de microcefalia por ZIKV foram registrados no Resp-Microcefalia apenas a partir de novembro de 2015, em meio à epidemia de ZIKV. A impossibilidade de acesso, pelo SUIBE, aos requerimentos de BPC/E87 e de E60 não concedidos devido ao óbito ou qualquer outra questão do requerente antes de completar todo o processo para a concessão dos benefícios pode ter acarretado um viés no dimensionamento das famílias em situação de vulnerabilidade socioeconômica com crianças com microcefalia por SCZ ou outras causas, inviabilizando uma análise mais precisa. Por fim, a impossibilidade de realizar o *linkage* entre os bancos utilizados resultou em uma análise descritiva ecológica, importante como primeiro levantamento acerca de um grave problema de saúde pública no país.

## Conclusões

O padrão de concessão do BPC/E87 apresentou um aumento superior a três vezes para crianças com microcefalia nascidas durante a Espin-ZIKV em comparação ao período anterior a este. A ausência de outros fatores responsáveis pelo aumento nas concessões de BPC/E87 por microcefalia sem causa especificada sugere que parte dessas concessões corresponderam a casos de SCZ, pois a *Lei nº 13.301/2016* determinou que as crianças com SCZ recebessem o BPC/E87 já existente. A *Lei nº 13.985/2020* orientou a migração desses casos para a E60, contudo a existência desse aumento injustificável de BPC/E87 ativos em crianças nascidas durante a Espin-ZIKV sugere que parte dessas crianças tenha sido mantida no benefício que já recebia.

A concessão de um percentual reduzido de E60 frente ao total de casos suspeitos de SCZ durante o período estudado, e com parte desses desprotegidos por lei específica, diante da associação entre vulnerabilidade econômica e infecções congênitas, demonstrou o desamparo dessas famílias, com a necessidade de melhor adequação das políticas públicas para esse grupo.

O estudo verificou que, mesmo com a redução da ocorrência de SCZ após Espin-ZIKV, as crianças com SCZ que nasceram após 2019 receberam BPC/E87, o que sugere uma proteção social ainda menor das crianças com microcefalia por outras causas.

A comparação entre as concessões de BPC/E87 em 2013 e 2021 mostrou redução significativa na concessão de BPC/E87 para crianças com microcefalia nascidas em 2021 em relação às nascidas em 2013; logo, não foi observada nenhuma curva de aprendizado dos profissionais de saúde sobre o acesso aos benefícios sociais para crianças com microcefalia após a Espin-ZIKV.

O presente estudo apresentou dados que indicam dificuldades quanto aos cuidados e proteção de crianças portadoras de microcefalia e suas famílias, e o quão vulneráveis elas ainda estão. As dificuldades para fazer a análise dos dados extraídos das plataformas utilizadas também indicam a necessidade de interligação das plataformas nacionais de informação para a criação de uma plataforma única que facilite a comunicação dos sistemas, auxilie as ações de cuidado à saúde e proteção social, melhore o acesso de benefícios à população e forneça informações com maior presteza para estudos e desenvolvimento de ações em saúde pública que visem o bem-estar da população.
